# Inflammatory Response Associated with West Nile Neuroinvasive Disease: A Systematic Review

**DOI:** 10.3390/v16030383

**Published:** 2024-02-29

**Authors:** Alessandro Pavesi, Giorgio Tiecco, Luca Rossi, Anita Sforza, Andrea Ciccarone, Federico Compostella, Sofia Lovatti, Lina Rachele Tomasoni, Francesco Castelli, Eugenia Quiros-Roldan

**Affiliations:** 1 Department of Clinical and Experimental Sciences, Unit of Infectious and Tropical Diseases, University of Brescia and ASST Spedali Civili di Brescia, 25123 Brescia, Italy; a.pavesi003@unibs.it (A.P.); g.tiecco@unibs.it (G.T.); l.rossi029@unibs.it (L.R.); a.sforza@unibs.it (A.S.); a.ciccarone@unibs.it (A.C.); federico.compostella91@gmail.com (F.C.); s.lovatti001@unibs.it (S.L.); francesco.castelli@unibs.it (F.C.); 2 Unit of Infectious and Tropical Diseases, ASST Spedali Civili di Brescia, 25123 Brescia, Italy; linatomasoni@yahoo.it

**Keywords:** West Nile, WNND, neuroinvasive disease, inflammation, cytokine, chemokine, arbovirus, One Health, review, systematic review

## Abstract

Background: West Nile virus (WNV) infection is a seasonal arbovirosis with the potential to cause severe neurological disease. Outcomes of the infection from WNV depend on viral factors (e.g., lineage) and host-intrinsic factors (e.g., age, sex, immunocompromising conditions). Immunity is essential to control the infection but may also prove detrimental to the host. Indeed, the persistence of high levels of pro-inflammatory cytokines and chemokines is associated with the development of blood–brain barrier (BBB) damage. Due to the importance of the inflammatory processes in the development of West Nile neuroinvasive disease (WNND), we reviewed the available literature on the subject. Methods: According to the 2020 updated PRISMA guidelines, all peer-reviewed articles regarding the inflammatory response associated with WNND were included. Results: One hundred and thirty-six articles were included in the data analysis and sorted into three groups (in vitro on-cell cultures, in vivo in animals, and in humans). The main cytokines found to be increased during WNND were IL-6 and TNF-α. We highlighted the generally small quantity and heterogeneity of information about the inflammatory patterns associated with WNND. Conclusions: Further studies are needed to understand the pathogenesis of WNND and to investigate the extent and the way the host inflammatory response either helps in controlling the infection or in worsening the outcomes. This might prove useful both for the development of target therapies and for the development of molecular markers allowing early identification of patients displaying an inflammatory response that puts them at a higher risk of developing neuroinvasive disease and who might thus benefit from early antiviral therapies.

## 1. Introduction

West Nile virus (WNV) is a single-stranded positive RNA (ssRNA+) virus belonging to the *Flaviviridae* family [1]. Its life cycle involves vertebrate species as the reservoir (birds) and incidental hosts (horses, alligators, humans), and invertebrate species as the vector of infection (mosquitos, usually *Culex* spp.) [2]. Consequently, in temperate regions of North America and Europe, infection with this arbovirus is typically seasonal, as it requires optimal conditions for its vector to reproduce: most infections are, indeed, temporarily clustered during the so-called “WNV transmission season” starting in June and ending in October [3]. During the past few years, WNV infection cases have risen in number and spread to new territories; the cause of this varying epidemiology is mainly to be found in climate change, which affects worldwide parameters such as temperature and humidity, making wider environments more suitable for mosquitos’ proliferation and contributing to a change in the migratory routes of birds [1,4,5]. Thus, WNV represents an optimal example of how a One Health approach should be integrated in tackling emergent infectious agents [6]. Less frequently documented ways of transmission include infected blood transfusion or solid organ transplant, and vertical transmission during pregnancy [1,7].

This pathogen causes an infection that is usually asymptomatic, but a self-limited mild febrile flu-like syndrome (known as West Nile fever, WNF) can be observed roughly in 20% of infected individuals, and 1 person out of 150 can develop a severe form of infection leading to central nervous system (CNS) involvement (known as West Nile neuroinvasive disease, WNND) [8,9]. To date, an older age is known to be the main risk factor for severe forms of infections. Patients aged over 65 years have a risk 16 times higher than younger individuals of developing severe disease, and those over 70 years of age with WNND have a risk of death 30–45 times higher than younger patients [10,11]. Other risk factors for neuroinvasive disease include the male sex (odds ratio [OR] = 1.3–1.6 for neuroinvasive disease) and various immunocompromising conditions (e.g., diabetes) [9,12,13]. As for other infections, immunologic and genetic determinants of severe WNV disease start to be considered [14,15]. Notwithstanding, the enormous interindividual clinical variability remains largely unexplained.

WNND encompasses a spectrum of neurological disorders: from the most frequent encephalitis (WNE, 50–70%) to meningitis (WNM, 15–35%), meningoencephalitis (WNME), and acute flaccid paralysis (AFP, 5–20%) [16]. The mortality associated with these syndromes is high (15–30%) and some patients can develop permanent neurological sequelae (motor, cognitive, or behavioral) [8,9,17].

Various therapeutic approaches have been tested but, to date, no specific treatment for WNV infection has been identified and the clinical management of patients relies on supportive care [18]. Likewise, prevention relies on measures as vector control or general protective provisions against mosquitos, as no vaccine against WNV has been licensed for human use [19,20]. National and international programs have been implemented to monitor the distribution of animal and human cases of WNV infection [3,21].

The virus pathobiology has been investigated. It is known that the virus is inoculated in humans via mosquito bite, then it locally replicates in the keratinocytes and Langerhans cells of the epidermis, and subsequently, it spreads to the draining lymph nodes and in the blood [1,22]. CNS involvement happens in few individuals through a hematogenous route (via multiple mechanisms; i.e., transudative, trans-endothelial, and “Trojan horse”) or a trans-neuronal route (via a retrograde axonal transport through the olfactive or peripheral nerves) [1,22]. Notwithstanding, only a few studies have described the difference in the inflammatory response among individuals developing an asymptomatic infection, WNF, or WNND [23].

To the best of our knowledge, no meta-analysis or systematic reviews are to date available regarding the inflammation profiles associated with the neuroinvasive disease. In the era of precision medicine and biological drugs, and considering the high mortality and morbidity associated with WNND, a better understanding of these aspects could be of use for clinicians to test and develop therapeutic strategies to reduce progression toward WNND at the early stages of infection (pre-emptive therapies) and to reduce inflammation-driven neurological damage during WNND (through biological therapies reducing the levels of target cytokines associated with neuroinflammation), thus improving outcomes of the infection.

Hence, we systematically review all the available literature describing the pattern of inflammation associated with WNV infection leading to CNS involvement in vitro, in animal models, and in humans, trying to assess the inflammatory profile of this neuroinvasive infection.

## 2. Methods

Our methods meet the Preferred Reporting Items for Systematic Reviews and Meta-Analysis (PRISMA) updated guidelines for systematic reviews stated in 2020 [24].

### 2.1. Eligibility Criteria

All randomized clinical trials (RCTs), prospective studies, retrospective studies, case series, or case reports, published in peer-reviewed medical journals, regarding WNV infection leading to CNS involvement were considered. Preclinical analyses, including in vitro and in vivo studies assessing the role of proinflammatory cytokine patterns in WNV infection, were also considered. We excluded articles in which the inflammatory response to WNV infection was not characterized, and papers published in non-English languages, pre-print or ahead-of-print analyses, reviews, short communications, letters to the editor, conference articles, viewpoints, and commentaries.

### 2.2. Information Sources and Search Strategy

An electronic search was employed to find the published articles that reported WNV infection with CNS involvement, and its inflammatory cytokine patterns, through the United States National Library of Medicine, PubMed (last accessed August 2023), MED-LINE (last accessed August 2023), PubMed Central, PMC (last accessed August 2023), and the Cochrane Controlled Trials (last accessed August 2023). References for this review were identified with the following research term combination: (“West Nile” OR “WNV”) AND (“inflammation” OR “cytokines” OR “cytokine” OR “markers” OR “marker”). No time window was applied to the search.

### 2.3. Selection and Data Collection Process

A team of seven resident doctors in Infectious and Tropical Diseases at the University of Brescia, Italy, read the abstract of each scientific work and independently selected the articles according to the established criteria (S.L., L.R., A.P., A.S., G.T., A.C., F.C.). A Professor in Infectious and Tropical Diseases at the University of Brescia, Italy (E.Q.-R.), and the Director of the Unit of Tropical Diseases of ASST Spedali Civili di Brescia, Italy (L.R.T.), revised the included and the rejected papers. In order to assess for eligibility by reading the full-text manuscript, the selected papers were split considering 3 categories of scientific analysis: in vitro (analyzed by G.T. and L.R.), in vivo (analyzed by A.S., A.C., F.C., and S.L.), and in humans (analyzed by A.P.). Each resident doctor read, collected, and synthesized the data for the articles assigned by using a detailed and dedicated database. Afterwards, a random reassignment of the articles was carried out and each doctor reviewed the data collected by colleagues. Disagreements were resolved by a joint discussion supervised by the aforementioned Professor and Director.

### 2.4. Definitions

For the purpose of this review, wild-type animals and mutated animals used normally as wild-type (C57BL/6, C57BL/6J, FVB/NJ, CD1, Swiss webster) were equally considered as “wild-type”. As regards the articles dealing with the inflammatory response in vivo, we considered data only from wild-type animals in which cytokine expression was analyzed in blood, cerebro-spinal fluid (CSF), or brain tissue samples. We also excluded data from animals vaccinated or treated. Additionally, due to the lack of a definition of the acute phase of WNV infection in humans, it was agreed that the “acute phase” would be defined as lasting for 90 days post-infection, as this is the time necessary for IgM (classically considered as a marker of acute infection) to disappear in most patients [25]. “Late phase” thus refers to any analysis performed following WNND, after more than 90 days post-infection.

### 2.5. Data Items

For each selected article, the following data were considered: first author, journal, year and country of publication, and type of article. In the dedicated database created for every group (in vitro, in vivo, and in humans), a column was warranted to any cytokine or chemokine cited in the analyzed articles. For every article, it was reported whether the molecule was found increased, decreased, or unchanged following WNV neuroinvasive infection. The method used for cytokine/chemokine testing was recorded.

In the case of:

- in vitro articles, the following items were additionally considered: the cellular line and viral strain used for the analysis, and viral replication data, including viral replication peak.
- in vivo articles, the following items were additionally considered: the animal species and viral strain used for the analysis, and the sample tested. Where possible, the entity and the timing of the cytokine/chemokine decreasing or increasing levels was specified.
- in human articles, the following items were additionally considered: the cohort dimension and the sample tested. The database was split into a first part regarding the analysis performed on patients’ sera (considering both the acute phase and the late phase of infection) and into a second part regarding the analysis performed on patients’ CSF. The control group used for the analysis in each article and the number of days post-infection on which the analysis was performed were specified.

### 2.6. Statistical Analysis

A descriptive analysis of the collected data was performed. No inferential analysis was carried out due to the wide heterogenicity of the included articles. Continuous variables with a Gaussian distribution are described using the mean value and standard deviation. Continuous variables with non-Gaussian distribution are described using the median value and interquartile range. Categorical variables are represented with their absolute and percentage frequency.

## 3. Results

### 3.1. Search Results and Study Selection

**Figure 1:**
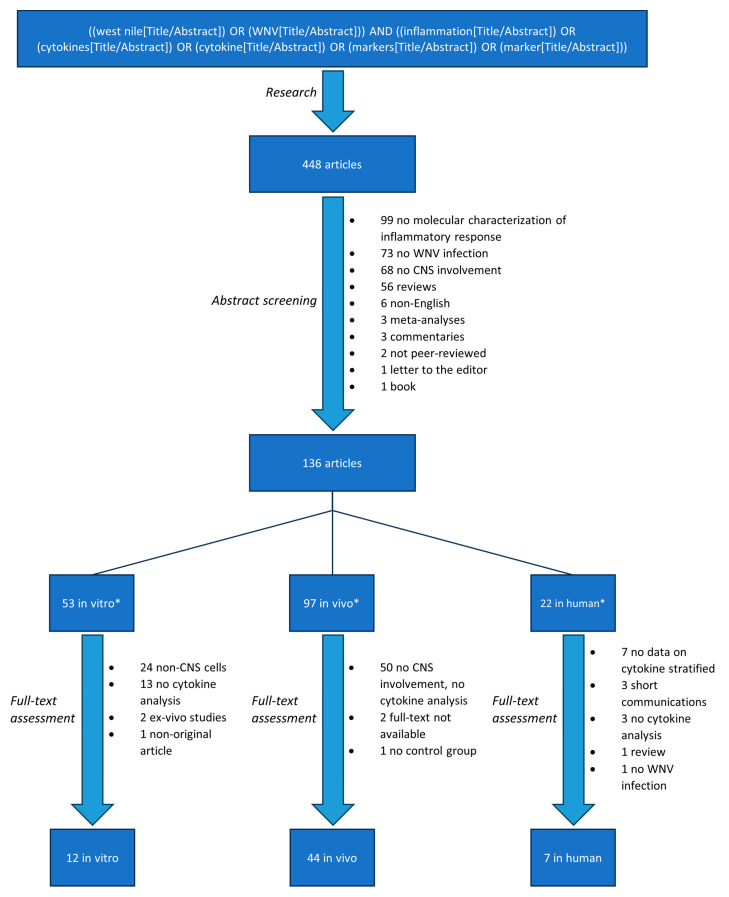
Search strategy and selection process. * Some articles were sorted into more than one group.

A total of 448 papers were identified through our search and screened for eligibility by reading their abstracts. Three hundred and twelve articles were excluded as they did not meet at least one eligibility criterion (Figure 1). The remaining 136 articles were divided into 3 groups (in vitro, in vivo, and in humans) and assessed for eligibility after a full-text analysis. A total of 53 articles were sorted into the in vitro group, 97 into the in vivo group, and 22 into the in human group. Following full-text analysis, a further 41, 53, and 15 articles were excluded from the in vitro, in vivo, and in humans groups, respectively (Figure 1). The remaining 12 articles included in vitro were experimental studies conducted on CNS cell cultures, which analyzed cytokine production during WNV infection. A total of 44 prospective studies were included for the in vivo data analysis, and 7 manuscripts (6 retrospective studies and 1 case series) were analyzed for cytokine/chemokine production during or following WNND in humans (Figure 1).

A list of the articles included in our review is reported in the following table (Table 1).

### 3.2. In Vitro Analysis

**Figure 2:**
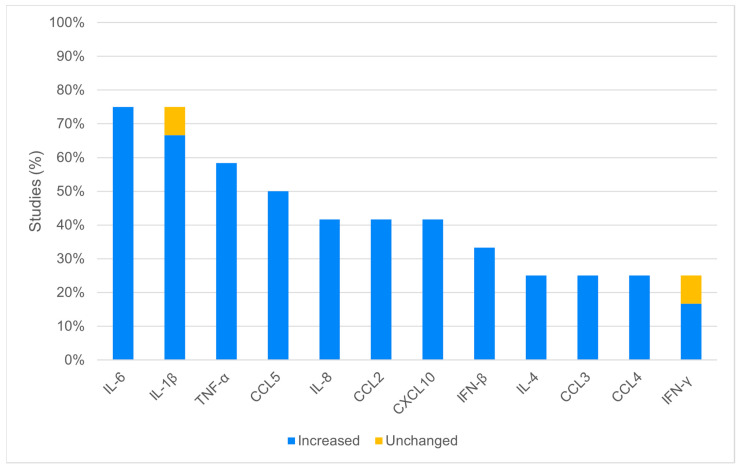
Expression of the most frequently in vitro analyzed cytokines: the figure reports the expression of the most analyzed cytokines in the included studies (at least 25.0% or 3/12). Not shown: cytokines analyzed in 2 (2/12, 16.7%) studies (IL-2, IL-10, IL-17, IL-18, IFN-α, and GM-CSF), and in only one (1/12, 8.3%) study (IL-1α, IL-1ra, IL-5, IL-7, IL-9, IL-12, IL-15, CXCL9, VEGF, FGF, PDGF-BB, and Eotaxin).

The most frequent virus strain was WNV NY99, used in 5 out of 12 studies (41.7%). In 8 (66.7%) studies, viral replication within CNS cells was analyzed, finding active replication in all the cell lines tested, even if with some differences. One study showed that microglial cells support lower WNV replication compared to neurons and astrocytes [31]. To characterize the inflammatory response, the included studies used qRT-PCR (5/12, 41.7%), immunoassays (2/12, 16.7%) or both (4/12, 33.3%). The inflammatory profile and cytokines involved were variably analyzed and are summarized in Figure 2. The cytokines most frequently increased during WNV infection were IL-6 (9/12, 75%), IL-1β (8/12, 66.7%), TNF-α (7/12, 58.3%), CCL5 (6/12, 50%), CCL2 (5/12, 41.7%), CXCL10 (5/12, 41.7%), and IL-8 (5/12, 41.7%) (Figure 2). In one study conducted on glial cell cultures (microglia/astrocytes), the levels of IL-1β and IFN-γ in infected cells were not increased compared with mock-infected controls [31].

In addition to the characterization of the inflammatory response, some studies have shown other interesting results. It was found that UV inactivation of WNV decreased both chemokine and cytokine production [31]. Additionally, Kumar M. et al. observed that apoptosis of infected cells increased in a dose- and time-dependent manner, and the use of anti-IL-1β and anti-TNF-α reduced the neurotoxic effects [26].

### 3.3. In Vivo Analysis

Of the included articles, 40 (90.9%) were on mice, 2 (4.5%) on horses, 1 (2.3%) on rabbits and 1 on primates. In 34 out of 44 analyzed articles (77.3%), the viral load was assessed in the CNS (CSF or brain samples), in 23 out of 44 (52.3%), in the blood, and only in 1 article it was sought in urine. The WNV strain mostly used was NY99 (23/44, 52.3%). The change in cytokine and chemokine expression was compared with either the corresponding wild-type uninfected or mock virus-infected animal. Three types of methods were used for cytokine and chemokine analysis: PCR (241/376, 64.1%), immunoassay (132/376, 35.1%), and BLAST (3/376, 0.8%). PCR was used to assess the gene expression, while immunoassays were employed for protein quantification.

**Figure 3:**
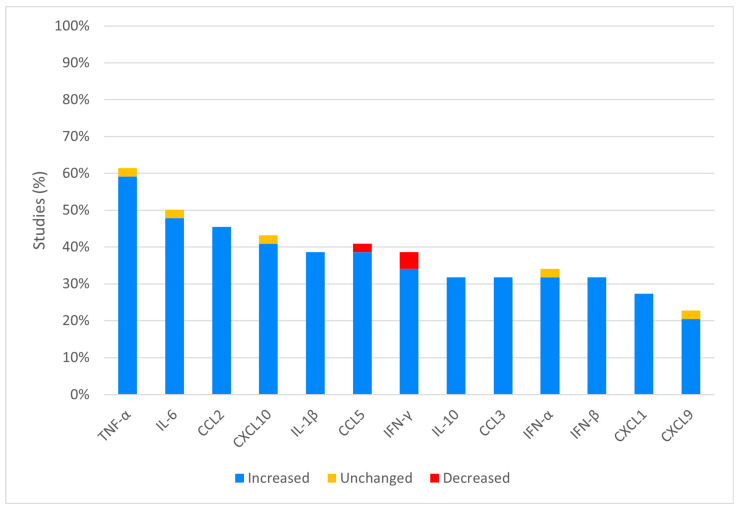
Expression of the most frequently in vivo analyzed cytokines: the figure reports the expression of the most analyzed cytokines in the included studies (at least 20.0% or 9/44). Not shown: cytokines analyzed in less than 9 studies (CCL4, IL-1α, CCL7, IL-12p40, IL-12, G-CSF, IL-4, IL-13, IL-17, CCL8, CCL11, CXCL2, CXCL12, CXCL13, IL-5, IL-7, IL-22, IRF-1, IRF-7, GM-CSF, IL-12β, IL-15, CXCL5, CXCL11, TGF-β, IFIT-1, M-CSF, IL-2, IL-9, IL-12p70, IL-18, CCL1, CCL12, CCL19, CCL20, CCL24, CCL25, CXCL8, CXCL16, ISG-15, MX-1, RIG-I, TRAIL, MIF, and Eotaxin).

The molecule most frequently analyzed was TNF-α (27/44, 61.4%), followed by: IL-6 (22/44, 50.0%), CCL2 (20/44, 45.5%), CXCL10 (19/44, 43.2%), CCL5 (18/44, 40.9%), IFN-γ and IL-1β (17/44, 38.6%), and IFN-α (15/44, 34.1%). The other mostly represented cytokines and chemokines are summarized in Figure 3. An increase in cytokine expression was found in most studies (351/376, 93.3%) (Figure 3). Whenever observed, the decrease in cytokine or chemokine levels was related to a downregulation of the related gene.

In 16 out of 44 articles (36.4%), it was possible to establish a temporal pattern of cytokine/chemokine growth. The increase in cytokine and chemokine levels was noted to occur earlier in blood than in the CNS. In blood, in most cases (5/16, 31.2%), the peak level was observed between day 2 and day 4 post-infection, followed by a decrease in concentration. Only in two studies the peak level was recorded at day 6 and day 10. In the CNS, in most cases (10/16, 62.5%), the peak level was observed between day 5 and day 8 post-infection. In two cases, it was recorded earlier (at day 2 and at day 4), and in one case, later (at day 25).

### 3.4. In Humans Analysis

Two hundred and sixty patients were analyzed for cytokine and chemokine production in serum and/or CSF, during or following WNND, in the included studies. The cytokine and chemokine levels were assessed mainly through antibody-based techniques (6/7, 85.7%).

**Figure 4:**
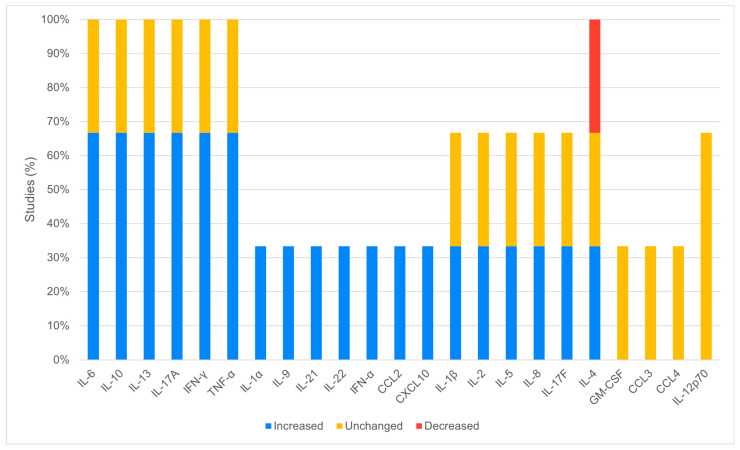
Expression of the analyzed cytokines in human serum samples during the acute phase of WNV infection: the figure reports the expression of the analyzed cytokines in the included studies. All cytokines are shown.

Three articles (3/7, 42.9%) analyzed the cytokine and chemokine levels in serum during the acute phase of infection. The molecules most frequently found to be increased were IL-6, IL-10, IL-13, IL-17A, IFN-γ, TNF-α (2/3, 66.7%), and IL-1α, IL-1β, IL-2, IL-4, IL-5, IL-8, IL-9, IL-17F, IL-21, IL-22, IFN-α, CCL2, CXCL10 (1/3, 33.3%). Only IL-4 was found to be reduced in one study [23]. Otherwise, the molecules were found to be unchanged with respect to the control group or were not tested (Figure 4). The control group was represented, in two cases, by healthy individuals not infected with WNV and, in one case, by cytokine and chemokine reference levels in serum.

Three articles (3/7, 42.9%) analyzed the cytokine and chemokine levels in serum during the late phase of infection. The mean time of serum sampling after WNND was 2319 ± 210 days. Variations from the control group were found for: IL-1β, IL-4 and CXCL10, which were found to be reduced in one study, respectively [79,80], and G-CSF, which was found to be increased in one article [77]. The control group was, in all cases, represented by patients previously infected with WNV but having not developed WNND.

Three articles (3/7, 42.9%) analyzed the cytokine and chemokine levels in CSF during the acute phase of infection. Zidovec-Lepej et al. found a reduction in IL-2, IL-4, IL-5, IL-17A, IL-17F, IL-21, TNF-α and an increase in IL-6 in the CSF of patients with WNND compared to their serum [23]. Constant et al. observed an increase in IFN-γ in the CSF of patients with WNV encephalitis with respect to patients with WNV meningitis [78]. Finally, Normandin et al. recorded an increase in IL-6 and IL-16 and a decrease in CCL-4 in CSF of patients with WNND compared with CSF of healthy subjects [81]. Globally, the molecules most frequently found to be increased in CSF were IL-6 (2/3, 66.7%), IL-16 (1/3, 33.3%), and IFN-γ (1/3, 33.3%).

## 4. Discussion

We reviewed the available literature data about the molecular profile of inflammation associated with WNND. Most of the literature included in this review is focused on immunity to experimental WNV infection both “in animals” and “in vitro”, and only seven papers studied the inflammatory response in natural conditions of WNND in humans.

In general, the included articles showed an increase in the levels of the investigated cytokines and chemokines.

Following WNV infection, as for other viral infections, an initial innate immune response involving pro-inflammatory cytokines is initiated and it is considered essential in the initiation and maintenance of inflammation for the viral replication control [83]. However, overexpression and continuous upregulation of inflammatory cytokines’ genes may be detrimental in some viral infections, including WNV, due to enhancing the severity of the infection and inflammation, leading to death, chronic or permanent morbidity and/or sequelae such as immunopathology [84]. Some of these cytokines may remain upregulated, even long after recovery from WNV infection [85]. Therefore, understanding the immune response during WNND could help in finding therapeutic options.

Of note, while necessarily for articles in vitro and in vivo it was not possible to assess cytokine and chemokine production at a distance from WNV infection, for the articles in humans it was decided to evaluate cytokine and chemokine production both during the acute and the late phase of infection (previously defined in the Methods as an evaluation after >90 days post-infection). The rationale behind this choice is that data regarding cytokines and chemokines at a later timepoint, compared between patients who did and did not experience neuroinvasive disease following WNV infection, may be informative about an immunologic higher susceptibility to WNND. Moreover, specific sequelae may be correlated—when observed—with a pattern of upregulation of specific cytokines and chemokines.

### 4.1. Increased Cytokines and Chemokines

Pro-inflammatory cytokines play a key role in the host immune response to infection and they include IL-1β, IL-8, IL-12, IL-17, IFN-γ, and TNF-α [86]. Among these, the most frequently found to be increased in our review was TNF-α (58.3% in vitro, 59.1% in vivo, and 66.7% in human serum during the acute phase of infection), followed by IL-1β (66.7% in vitro, 38.6% in vivo, and 33.3% in human serum during the acute phase of infection) and IFN-γ (16.7% in vitro, 34.1% in vivo, 66.7% in human serum during the acute phase of infection, and 33.3% in human CSF during the acute phase of infection). These pro-inflammatory cytokines are well known for exerting protective effects during infection, but they can also be detrimental to the host as they can induce inflammation-related damages, as it has been described for other pathogens [84,87].

Interleukin-6 (IL-6) is a pleiotropic cytokine, which possesses both pro-inflammatory and anti-inflammatory properties, secreted by multiple cells (B and T lymphocytes, monocytes, fibroblasts, and endothelial cells) [86]. IL-6 showed an increase in 75% of articles in vitro, in 47.8% of articles in vivo, in 66.7% of articles analyzing human serum during WNND, and in 66.7% of articles analyzing human CSF during WNND. Therefore, it represents the most over-produced cytokine during WNV infection with CNS involvement in our review. Particularly interesting results are reported in some human studies: for instance, Zidovec-Lepej et al. described an increase in IL-6 levels in CSF, while they already found it increased in serum [23]. Moving on from this observation, we can speculate that IL-6 is central both in the process of WNV-induced systemic inflammation and in neuroinflammation. Intriguingly, the only article in which IL-6 was not found to be increased in CSF is the one comparing WNM samples to WNE samples: we may thus hypothesize that the cytokine is, actually, equally incremented in these two clinical manifestations of WNND [78]. These findings appear coherent with what is already known from the literature about IL-6 pivotal role in the course of other infections [88,89].

Anti-inflammatory cytokines serve to inhibit inflammation and suppress immune cells, and they include IL-4, IL-10, IL-11, IL-13, IL-1 receptor antagonist (IL-1ra), and TGF-β [86]. In our review, they were less frequently found to be increased compared to pro-inflammatory cytokines. An exception is represented by IL-10 and IL-13, which were found to be increased in 66.7% of studies assessing human serum cytokine production during the WNND acute phase. The observation of higher levels of IL-10 is in line with earlier studies, which already described a correlation between its increase and worse outcomes of infection [90].

IFN-α and IFN-β belong to the family of type I IFN. These molecules usually increase following viral infection as they mediate early antiviral responses [91]. An impairment in their function, as observed in the context of the production of autoantibodies anti-type I IFNs, has been shown to determine worse outcomes following WNV infection [14,15]. We have observed an increase in IFN-α or IFN-β in about one-third of the included articles, with almost the same proportions found in studies in vitro, in vivo, and in humans. We could speculate that the reason for this modest proportion of articles showing an increase in type I IFN reflects the onset of serious disease in patients with limited capacity to produce these molecules, as previously reported by other investigators [92]. A second explanation may lie in the sample timing: Zidovec-Lepej et al. and Leis et al. tested sera for cytokines and chemokines 13 and 56 days post infection, respectively, while type I IFN response happens usually earlier following viral infection [23,82].

Chemokines are chemotactic cytokines that control the migration and positioning of immune cells in tissues and are critical for the function of the immune system [93]. In our review, we found most frequently increased CCL2 (41.7% in vitro, 45.5% in vivo, and 33.3% in human serum during the acute phase of infection), CCL5 (50% in vitro, 38.6% in vivo), and CXCL10 (41.7% in vitro, 40.9% in vivo, and 33.3% in human serum during the acute phase of infection). Our findings are coherent with what is already known from the pre-existing literature regarding the activity of these molecules during infectious diseases [94,95,96]. Indeed, CCL2 (MCP-1) is involved in inflammatory monocyte trafficking, CCL5 (RANTES) is involved in macrophage and NK cell migration as well as in T-cells-DC interactions, while CXCL10 (IP-10) is involved in the Th1 response and trafficking of Th1, CD8, and NK cells [93].

### 4.2. Decreased Cytokines and Chemokines

We expected to record mainly an increase in the levels of the investigated cytokines and chemokines. Therefore, whenever the articles reviewed displayed decreased levels, an explanation for such an observation was sought by carefully reading the results and the discussion and by trying to interpret the data in light of the current evidence available from the literature.

Cheeran et al. did not observe in glial cell cultures an increase in IL-1β and IFN-γ in infected cells compared with mock-infected controls [31]. While the authors did not provide a specific explanation for this observation, we can hypothesize that this result is secondary to the cellular line and technique, which were different compared to the other studies. Also, Uddin et al. described a decrease in IFN-γ in the CNS of experimentally WNV-infected horses but did not provide an explanation for their observation [40]. Similarly, a reduction in the IFN-γ, IL-2, and CCL5 levels in the brain of WNV-infected mice was neither discussed by Peña et al., nor by Sabouri et al. [49,70]. While, to the best of our knowledge, no work has specifically addressed the reasons why a decrease in IFN-γ is observed during WNV infection, some data can be drawn from the study of Senft et al., who described the ability of another virus (RSV) to inhibit IFN-γ-inducible transcriptional activation [97].

A decrease in the IL-4 levels was observed in human serum samples by Zidovec-Lepej et al. during the acute phase of infection, suggesting that the cytokine does not play an important role in the immune response to WNV [23]. On the other hand, Qian et al. described lower basal levels of IL-4 in the serum of patients who had experienced WNND compared to the serum of patients previously infected with WNV without neuroinvasive disease, thus hinting at an association between reduced serum basal levels of IL-4 and a more ominous form of WNV infection [79]. Therefore, we can speculate that a lower production of IL-4, a Th2 anti-inflammatory cytokine, is normal initially, as the infection is being countered with the production of mainly pro-inflammatory cytokines. Notwithstanding, we further speculate that the inability to produce adequate levels of IL-4 correlates with a higher probability of developing neuroinvasion. Indeed, it is known that the anti-inflammatory cytokines serve the key purpose of limiting the injurious effects of an uncontrolled inflammatory response: without sufficient IL-4 levels, the pro-inflammatory cytokines damage the BBB, thus facilitating WNV entry into the CNS. To the extent of what we know, the data are contrasting about the kinetics of IL-4 in humans following viral infection and they appear to be virus-specific [98,99]. Nevertheless, Rhodes et al. observed delayed IL-4 production during *M. bovis* infection in cattle, similarly to what we have hypothesized [100]. Additionally, Qian et al. also suggested a role for the reduced serum levels of IL-1β and CXCL10 in enhancing host susceptibility to more severe forms of WNV infection [80]. IL-1β is a key pro-inflammatory cytokine, and CXCL10 is a chemokine produced in response to IFNγ; therefore, their lack can result in an insufficient pro-inflammatory response to WNV, necessary to limit virus replication. Moving to other interesting results, the cytokines that were described by Zidovec-Lepej et al. as reduced in CSF during the acute phase of WNV infection have to be considered with respect to the control group used: paired sera samples [23]. Therefore, this study highlights the peripheral and central differential profiles of inflammation during WNND, with most cytokines mainly produced in serum. Finally, Normandin et al. did not discuss the evidence of a reduction in CCL4 in the CSF of patients with WNND compared to healthy controls, as their study focused rather on SARS-CoV-2 infection [81].

### 4.3. Study Strengths and Limitations

The findings of this systematic review should be seen in light of some limitations. At first, the heterogeneity of the studies included (which considered different panels of cytokines and chemokines), in the absence of methods to assess the risk of bias or certainty in the body of evidence, restricted our review to a descriptive analysis. For this reason, for instance, it was impossible to assess the impact of the virus lineage, the cell lines tested, and the animal models used on the inflammatory profile observed. Moreover, the cytokines’ levels were described only as increased, reduced or unchanged, without describing the entity of the variation, due to the wide heterogeneity in the methods of quantification and in the reference levels used. Furthermore, due to the lack of normal values of cytokines and chemokines in CSF, and due to the ethical issues linked to the possibility of performing LPs on healthy individuals, the control group for patients’ CSF was profoundly heterogeneous. Finally, it would be interesting to identify the cell types responsible for the production of the cytokines and chemokines detected, but this information was lacking in most of the included studies.

Notwithstanding, our work has some strengths. Foremost, to the best of our knowledge, it is the first review systematically diving into data regarding inflammation during neuroinvasive WNV infection, simultaneously investigating the inflammatory profiles in cell cultures, in animals, and in human subjects. Secondarily, we described not only increases in cytokine and chemokine production but also provided information about those molecules occasionally found unchanged or reduced. Moreover, we recorded any additional information regarding laboratory (e.g., type of system used for analysis, time of variations) and clinical (e.g., inflammatory profiles acutely and at distance) features that might prove useful for future studies.

In order to identify possible therapeutic targets, the protective or deleterious role of the markers presented could be further investigated, particularly through studies involving knock-out animal models if available.

## 5. Conclusions

This review has highlighted the small quantity and heterogeneity of information about the pathogenesis of WNND in various host species. IL-6 and TNF-α are key pro-inflammatory cytokines involved in the control of many viral infections, including WNV, but they also display an increase during WNND and appear as candidates for the cytokine-induced burden of illness. Interferons (IFNs) are necessary for the initial restriction of viral replication, and a reduced production of these molecules has been correlated with worse outcomes of infection.

Although WNV continues to spread and cause significant morbidity and mortality across the world, funding and research are scarce [101]. Currently, only 16 trials are registered in clinicaltrial.gov (9 for vaccines and 7 for therapies) [102]. With the current trend in climate change and aging of the population, the prevalence of WNND is expected to increase and its clinical management should use a multidisciplinary integrated approach involving virologists, immunologists, and infectious diseases specialist professionals. Also, further efforts are needed to understand the pathogenesis of WNV, focusing on the severe forms, in various animal species and humans. Moreover, there is an urgent need for effective treatments for limiting the viral replication phase in the high-risk groups to avoid clinical progression to severe forms, as currently available for SARS-CoV-2 infection [103]. Finally, it is important to develop effective and safe vaccines to control the disease in various animal species and humans.

## Figures and Tables

**Table 1:** Summary table of the in vitro, in vivo, and in humans articles.

**Articles In Vitro**							
**Author**	**Ref. N**	**Cellular Line or Technique**	**WNV Strain**	**Increased Cytokines**		**Decreased Cytokines**	**Unchanged Cytokines**
Kumar M	[26]	Transformed human neuroblastoma (SK-N-SH)	WNV NY99	TNF-α, IL-1β, IL-6, IL-8		NA	IL-18
**Constant O**	[27]	Human brain-like endothelial cells (hBLEC)	WNV 3125/France/2018	TNF-α, IL-1β, IL-4, IL-6, IL-8, IL-17, CCL2, CCL3, CCL4, CCL5, CXCL10, IFN-α, IFN-β, GM-CSF		NA	NA
Verma S	[28]	Human brain cortical astrocytes (HBCA)	WNV NY99	IL-1β, IL-6, IL-8		NA	NA
Huang B	[29]	Transformed human neuroblastoma (SK-N-SH)	WNVKUN (MRM16) + NSW2012	IL-2, CCL2, CCL5, CXCL10, IFN-β		NA	NA
Bhide K	[30]	Human brain microvascular endothelial cells (HBMECs)	WNV Goshawk	IL-1β, IL-6, CCL2, CCL5, CXCL10		NA	NA
Cheeran MC	[31]	Human glial cell cultures (microglia/astrocytes)	WNV NY99	TNF-α, IL-6, IL-8, CCL2, CCL3, CCL4, CCL5, CXCL10		NA	IL-1β, IL-10, IFN-α, IFN-γ
Nelson J	[32]	Human neural stem cell (hNSC)-derived neuron/astrocyte co-cultures	WNV NY99	TNF-α, IL-1β, IL-1ra, IL-2, IL-4, IL-5, IL-6, IL-7, IL-8, IL-9, IL-12, IL-15, IL-17, IL-18, CCL3, CCL4, CCL5, CXCL9, CXCL10, IFN-γ, GM-CSF, VEGF, FGF, PDGF-BB, Eotaxin		NA	NA
Zhang H	[33]	Human glial cell line (U251)	WNV NY99	TNF-α, IL-1β, IL-6		NA	NA
Durrant DM	[34]	Mice CNS tissue	WNV 3000.0259	IL-1α, IL-1β		NA	NA
Getts DR	[35]	Mice CNS tissue	WNV Sarafend	TNF-α, IL-6		NA	NA
Stonedahl S	[36]	Mice brain slice cultures	WNV TX02	Il-4, IL-6, IL-10, CCL2, CCL5, IFN-β, IFN-γ		NA	NA
**Daniels BP**	[37]	Brain microvascular endothelial cells (BMECs)	WNV 3000.0259	TNF-α, IL-1β, IFN-β		NA	NA
**Articles in vivo**							
**Author**	**Ref. N**	**Species**	**WNV Strain**	**Increased Cytokines**		**Decreased Cytokines**	**Unchanged Cytokines**
Patel S	[38]	Mouse	WNV ITA09	TNF-α, IFN-γ		NA	NA
Luo H	[39]	Mouse	WNV NY99	TNF-α, IL-12, CCL2, CCL4, CXCL10		NA	NA
Jasim Uddin M	[40]	Horse	WNV NSW2011	IFN-α, CXCL10, ISG15, IRF7, IL-22		IFN-γ	NA
Natekar J P	[41]	Mouse	WNV NY99, WNV Eg101	IFN-α		NA	NA
Constant O	[27]	Mouse	WNV-3125/France/2018	TNF-α, CCL5		NA	IL-1β
Krause K	[42]	Mouse	WNV NY99	TNF-α, IFN-α, IFN-γ, IFN-β, IL-12p40, IL-6, CCL2, CCL4, CXCL10, IL-10 (blood sample), IL-1β, IL-1α, IL-5, GM-CSF, CCL5, CXCL2, CXCL9, IL-13, G-CSF, M-CSF, CXCL1, CCL3, IL-12p70, Eotaxin		NA	IL-10 (CNS sample)
Saxena V	[43]	Mouse	WNV H8912	IFN-β, IL-10, IL-1β		NA	TNF-α, IFN-α, IL-6
Rothan H A	[44]	Mouse	WNV NY99, WNV Eg101	TNF-α, IFN-α, IFN-γ, IL-6, CCL2, CXCL10, IL-1α, CXCL9		NA	IL-5, G-CSF
Wang P	[45]	Mouse	WNV-2741	TNF-α, IFN-β, IL-6, CXCL1, CXCL5, IL-22		NA	NA
Welte T	[46]	Mouse	WNV NY99	IL-10, IL-17		TGF-β	NA
Michlmayr D	[47]	Mouse	WNV NY99	TNF-α, IFN-α, IFN-β, IL-6, CCL2, CCL4, CCL10, TGF-β, IRF7, IL-1β, CCL5, CXCL2, CXCL9, CCL11, CXCL1, CCL7, CCL8, CCL3, RIG-I		NA	CXCL12
Durrant DM	[48]	Mouse	WNV NY99	CCL2, CCL4, CXCL10, IL-1β, CCL5, CXCL9, CXCL12, CCL7, CCL3		NA	NA
Peña J	[49]	Mouse	WNV NY99	IL-12p40, IL-12, IL-6, CCL2, IL-10, IL-1α, GM-CSF, CCL5, IL-13, G-CSF, CCL11, CXCL1		IFN-γ, IL-2	NA
**Durrant DM**	[34]	Mouse	WNV NY99	IFN-α, IFN-β, IL-1β, IL-1α		NA	NA
Seitz S	[50]	Mouse	WNV NY99	IFN-γ, CCL2, CXCL10, CCL5, CXCL9, CCL7, CCL3		NA	NA
Maximova OA	[51]	Non-human primates	WNV NY99	TNF-α, IFN-γ, CCL2, CXCL10, CXCL11, CXCL8, CCL5, CXCL1, CXCL13, CCL8, CCL3		NA	NA
Clarke P	[52]	Mouse	WNV NY99	TNF-α, CCL2, CXCL10, CXCL11, CCL5, CXCL9, CXCL13, CCL7, CCL3, CCL12		NA	NA
Rosen SF	[53]	Mouse	WNV-NS5-E218A	CXCL16		NA	NA
Getts DR	[35]	Mouse	NA	TNF-α, IL-6		NA	NA
Clarke P	[54]	Mouse	WNV NY99	TNF-α, IFN-β, IL-6, CCL2, CXCL10, IL-4, IL-10, IFIT1, IRF1, CCL5		NA	NA
**Quick ED**	[55]	Mouse	WNV NY99	TNF-α, IFN-α, IL-6, CCL2, CXCL10, IL-4, IL-10, IRF1, IL-1β, IL-7, CCL5, IL-13, CCL7, CCL3		NA	NA
**Quick ED**	[56]	Mouse	WNV NY99	TNF-α, IL-6, CCL2, CXCL10, CCL5, CXCL1, CCL3, TRAIL		NA	NA
**Garber C**	[57]	Mouse	WNV NY99, WNV-NS5-E218A	IL-1β		NA	NA
Wang T	[58]	Mouse	WNV-2741	TNF-α, IFN-α, IFN-β, IL-12, IL-6		NA	NA
Arjona A	[59]	Mouse	WNV-2741	TNF-α, IFN-α, IL-12, IL-6, IL-1β, MIF		NA	NA
Lim SM	[60]	Mouse	WNV NY99	TNF-α, IFN-γ, IL-12b, IL-12, IL-6, CCL2, CCL3, CCL4, CCL5, CCL8, CXCL10, IL-10, IL-1β, CXCL2, CXCL9, CCL11, CXCL1, CXCL13, CCL25		CXCL12	IL-18, IL-23, IL-17, CCL1, CCL20, CCL24
**Daniels BP**	[37]	Mouse	NY-2000	TNF-α, IFN-β		NA	NA
Wang P	[61]	Mouse	NA	TNF-α, IFN-α, IL-6		NA	NA
Town T	[62]	Mouse	WNV-2741	IL-12b, IL-12p40, IL-23		NA	NA
Bai F	[63]	Mouse	WNV-2741	IFN-γ, IL-10		NA	NA
Kumar M	[64]	Mouse	Eg101, WNV NY99	TNF-α, IFN-γ, IL-6, CCL2, CCL4, CXCL10, IL-10, IL-1β, IL-1α, IL-5, IL-7, GM-CSF, CCL5, CXCL2, CXCL9, IL-15, IL-13, IL-17, G-CSF, M-CSF, CCL11, CXCL1, CXCL5, CCL3		NA	NA
Ramos HJ	[65]	Mouse	TX 2002-HC	IFN-β, IL-6, CCL2, IL-1β		NA	NA
**Kumar M**	[66]	Mouse	Eg101, WNV NY99	CCL4, CXCL10, IL-1α, IL-1β, IL-7, CCL5, CXCL1, CXCL12, CXCL13, CCL7, CCL8, CCL3, CCL19		NA	NA
Kumar M	[67]	Mouse	WNV NY99	TNF-α, IFN-γ, CCL2, CXCL10, IL-10, IL-1β, CCL5, CXCL1, CCL3		NA	NA
Kumar M	[68]	Mouse	WNV NY99	TNF-α, IFN-γ, CCL2, CXCL10, IL-1β, CCL5, CXCL9, G-CSF, CXCL1		NA	NA
Kumar M	[69]	Mouse	WNV NY99	TNF-α, IFN-γ, IL-6, CCL2, IL-1β, CCL5, CXCL1, CCL3		NA	NA
Sabouri AH	[70]	Mouse	Eg101	TNF-α, IFN-α, IL-6, CCL2, CCL3		CCL5	CCL4, CXCL10, CXCL9
Daffis S	[71]	Mouse	WNV 3004.19.00	IFN-α, IFN-β		NA	NA
Stonedahl S	[36]	Mouse	TX02	IFN-γ, IFN-β, IL-6, CCL2, IL-4, IL-10, MX1, IFIT1, IRF1, CCL5		NA	NA
**Paul AM**	[72]	Mouse	WNV-2741	IFN-α, IFN-β, IRF7		NA	NA
Xie G	[73]	Mouse	NS4B-P38G	TNF-α, IL-12p40, IL-10, IL-1β, IL-17, IFN-γ, IL-6		NA	NA
**Bourgeois MA**	[74]	Horse	WNV NY99	IL-15, IL-9, IL-22		NA	NA
**Suen WW**	[75]	Rabbit	NSW2011, TX8667	TNF-α, IFN-γ, IFN-β, IL-6, CXCL10, IL-4, IL-10		NA	NA
Acharya D	[76]	Mouse	WNV-2741	TNF-α, IFN-γ, IFN-α, IFN-β, IL-12p40, IL-6, IL-10		NA	NA
**Articles in humans**							
**Author**	**Ref. N**	**Number of Patients**	**Sample and Timing of Cytokine Analysis**	**Increased Cytokines**	**Decreased Cytokines**	**Unchanged Cytokines**	**Reference Group**
Lino A	[77]	29	Serum, late phase	G-CSF	NA	NA	Subjects with a history of asymptomatic WNV infection
Constant O	[78]	58	Serum, acute phase CSF, acute phase	*Serum*: IL-1α, IL-1β, IL-4, IL-6, IL-8, IL-10, IL-13, IL-17A, IFNα, IFNγ, CCL2, CXCL10 *CSF*: IFNγ	NA	*Serum*: IL-12p70, TNFα, GM-CSF, CCL3, CCL4 *CSF*: IL-1α, IL-1β, IL-4, IL-6, IL-8, IL-10, IL-12p70, IL-13, IL-17A, IFNα, TNFα, GM-CSF, CCL2, CCL3, CCL4, CXCL10	*Serum*: healthy individuals *CSF*: meningitis vs. encephalitis
Zidovec-Lepej S	[23]	60	Serum, acute phase CSF, acute phase	*Serum*: IL-2, IL-5, IL-6, IL-9, IL-10, IL-13, IL-17A, IL-17F, IL-21, IL-22, IFNγ, TNFα *CSF*: IL-6	*Serum*: IL-4 *CSF*: IL-2, IL-4, IL-5, IL-17A, IL-17F, IL-21, TNFα	*CSF*: IL-9, IL-10, IL-13, IL-22, IFNγ	*Serum*: healthy individuals *CSF*: paired serum
Qian F	[79]	59	Serum, late phase	NA	IL-4	NA	Subjects with a history of asymptomatic WNV infection
Qian F	[80]	49	Serum, late phase	NA	IL-1β, CXCL10	NA	Subjects with a history of asymptomatic WNV infection
Normandin E	[81]	4	CSF, acute phase	IL-6, IL-16	CCL4	IL-7, IL-8, IL-15, CCL2, CCL22	Healthy individuals
**Leis AA**	[82]	1	Serum, acute phase	TNFα	NA	IL-1β, IL-2, IL-4, IL-5, IL-6, IL-8, IL-10, IL-12p70, IL-13, IL-17A, IL-17F, IFNγ	Cytokine reference levels

For every article in vitro, the following items are described: author, reference number, type of article, cellular line or technique, West Nile virus strain, increased or decreased or unchanged cytokines. For every article in vivo, the following items are described: author, reference number, type of article, animal species, West Nile virus strain, increased or decreased or unchanged cytokines. For every article in humans, the following items are described: first author, reference number, type of article, sample size, sample used for cytokine analysis and timing, increased, or decreased or unchanged cytokine levels, and reference group. Abbreviations: CCL (C–C motif chemokine ligand), CNS (central nervous system), CSF (cerebro-spinal fluid), CXCL (C–X–C motif chemokine ligand), FGF (fibroblast growth factor), G-CSF (granulocyte colony-stimulating factor), GM-CSF (granulocyte monocyte colony-stimulating factor), IFIT (interferon-induced proteins with tetratricopeptide repeats), IFN (interferon), IL (interleukin), IRF (interferon regulatory factor), M-CSF (monocyte colony-stimulating factor), NA (not applicable), PDGF (platelet-derived growth factor), TNF (tumor necrosis factor), VEGF (vascular endothelial growth factor), WNV (West Nile virus).

## Data Availability

No new data were created or analyzed in this study. Data sharing is not applicable to this article.
